# Case of Well-Marked Purpura Hæmorrhagica, Successfully Treated by the Evacuant Plan

**Published:** 1825-03

**Authors:** Wm. Belcher

**Affiliations:** Licentiate of the Royal College of Surgeons in Ireland.


THE LONDON

### Medical and Physical Journal.

N° 3 OF VOL. LIII.] MARCH, 1825. [N° 313.

For many fortunate discoveries in medicine, and for the detection of numerous errors, the world is indebted to the rapid circulation of Monthly Journals; and there never existed any work, to which the Faculty, in Europe and America, were under deeper obligations, than to the Medical and Physical Journal of London, now forming a long, but an invaluable, series.—RUSH.

### ORIGINAL COMMUNICATIONS,

### SELECT OBSERVATIONS, &c.

ART. I.—*Case of well-marked Purpura Hæmorrhagica, successfully treated by the Evacuant Plan.* By WM. BELCHER, M.D. Licentiate of the Royal College of Surgeons in Ireland.

MASTER E—, ætatis three years and a half, previously a healthy child, was suddenly taken ill, on Sunday afternoon, November 21st, 1824, with lassitude, faintness, pains in the lumbar regions and joints, indisposition to make the slightest exertion, accompanied by nausea. These symptoms were soon followed by large and repeated discharges of dark fluid blood from the nose, mouth, and anus; the stools exhibiting no trace of fæcal matter. Considerable apparent debility was observable. Shortly afterwards, dark purple blotches, varying in size, developed themselves on the trunk, arms, and thighs. These symptoms continued unabated until the morning of Tuesday, the 23d, when I saw the patient; and, in addition to the before-mentioned symptoms, observed the tongue covered with a brown fur, interspersed with purple spots; the mucous membrane of the mouth and fauces appeared covered with them; the pulse was accelerated to 110, though feeble and compressible; the conjunctivæ, lips, apex and alæ nasi, were blanched; there was general retrocession of heat from the surface, which felt cold and constricted. He had all the symptoms of extreme debility, with the exception of the furred tongue and quick pulse. The debility at this moment appeared to me so urgent, and his friends so importunate for stimuli, that I thought it necessary to precribe small quantities of port-wine negus; and, as I had, with few exceptions, seen this obscure disease treated by stimulants, tonics, and nutrient diet, I ordered the mixture of infus. rosar., tinct. cinchon., with diluted sulph. acid., a dose every two hours; an anodyne enema of mucilag. amyli, with tinct. opii, to be immediately thrown up; and chicken-broth to be taken during the day.

I had soon ample reason to alter my plan to that of evacuants, as I was called in a hurry, at eight o'clock the same afternoon; the messenger stating the child was much worse. On my arrival, I found that his friends had adhered strictly to my directions, and that all the symptoms were aggravated: the bloody discharges had been more profuse and frequent; the pulse had risen to 140, and had become fuller and harder; considerable heat and dryness of the surface, flushing of the face, grinding of the teeth, strabismus, convulsive actions of the muscles of the lips, face, and forehead, were observable. I ordered him immediately into a warm bath at 100° Fahrenheit, and to remain until he became faint. He had been very few minutes in it, when the convulsive actions of lips, face, and forehead, grinding of teeth, and strabismus, ceased; a copious perspiration burst forth on the forehead and face, and he became faint. He was then well dried, and put into bed; a bolus of calomel and rhubarb given, washed down by repeated draughts of warm whey; a terebinthinate enema ordered to be immediately thrown up, and repeated every two hours, until copious intestinal discharges were produced.

Wednesday morning, the 24th.—Found him much better; pulse, tongue, and skin, relieved; bloody discharges and blotches still undiminished.—Continue the bath, bolus, and injections.

Thursday, 25th.—He has been much improved since yesterday. The oral and nasal hemorrhages have ceased; the purple spots on the tongue and inside of the mouth have disappeared; the others, on the trunk and extremities, have become of a pale-brown hue; and traces of fæcal matter are to be seen in the stools.—Continue the bath, bolus, and injections.

Friday, 26th.—Every symptom has improved: the blotches are gradually disappearing, as the stools become more natural; there is very little blood in them to-day.—Intermit the bath; continue the bolus and injections.

Saturday, 27th.—Further improvement in every respect. Stools now natural, with very slight traces of blood in them; the greater number of the blotches are gone; a few remain, and they are of a pale-brown colour.—Continue the bolus and injections.

In two days afterwards, the blotches had all disappeared, the stools had become natural, and the patient convalescent, though considerable debility remained.

*Remarks.*—The successful issue of this well-marked case of purpura by the evacuant plan, has made a strong impression on my mind of its superiority over the older and more favourite method by tonics and stimulants. If I had continued the treatment I commenced with, I am confident I would have lost my patient. Dr. Harty, of Dublin, has been successful in many well-marked cases, by active and frequent doses of calomel and jalap, after witnessing the death of a patient treated by nutritive diet and tonic medicines: his practice is detailed in the Edinburgh Medical and Surgical Journal, for April 1813. In the above case, the warm bath appeared to be a valuable addition, as the skin, pulse, disposition to convulsion, and general feelings, were much relieved after its first trial. In those cases, as soon as the hepatic, intestinal, and cuticular secretions, are restored, the blotches gradually disappear. The mercurial purgatives, assisted by the terebinthinate injections, were of singular benefit in restoring the hepatic and intestinal secretions. I cannot too strongly speak of the utility of the oleum terebinthinæ in injections, not only in this case, but in other acute diseases, where the indication is to free the intestinal canal, and restore its healthy secretion. I generally combine it with sulphat. magnesiæ, which appears to add to its powerful and beneficial effects: in this way, it also promotes the urinary secretion.

The late Dr. Parry, of Bath, recommends venesection in this singular disease. He met with two cases, one in a lady, the other in an officer, where the concomitant pyrexia was considerable; and the blood drawn exhibited a tenacious and firm coagulum, with the sizy coat. In plethoric adults, venesection doubtless is a powerful remedy; but in children I question its utility, though the pulse may indicate it. Free purgation, accompanied by the debilitating effects of warm baths, in this case answered the purpose, without inducing that degree of debility which is consequent to general blood-letting, and which in this disease, in children, would not be unattended with danger. Even if venesection appeared to the practitioner the "unicum remedium," I much doubt if the patient's friends would allow it: on the contrary, they will importune you to give him wine and nutriment. If depletions were necessary, I should prefer local to general; leeches to the right hypochondrium, epigastrium, or in the neighbourhood of the anus, to relieve hepatic and intestinal congestion. Dr. Stoker, of Dublin, in his late observations on purpural effusions, recommends cautious blood-letting, antimonials, mercurial purgatives, and blisters to the right hypochondrium; as he conceives, and with good reason, that an intimate connexion exists between this disease and hepatic functional derangements.

In conclusion, I have only to add, that, in every future case which I meet with, I shall try the evacuant plan, disregarding debility, which, in the generality of cases, is more ideal than otherwise.

*Bandon; November 3d,* 1824.

